# Different tau fibril types reduce prion level in chronically and *de novo* infected cells

**DOI:** 10.1016/j.jbc.2023.105054

**Published:** 2023-07-15

**Authors:** Luigi Celauro, Anna Burato, Marco Zattoni, Elena De Cecco, Marco Fantuz, Federico Angelo Cazzaniga, Edoardo Bistaffa, Fabio Moda, Giuseppe Legname

**Affiliations:** 1 Department of Neuroscience, Laboratory of Prion Biology, Scuola Internazionale Superiore di Studi Avanzati (SISSA), Trieste, Italy; 2 Fondazione per la Ricerca Biomedica Avanzata VIMM, Padova, Italy; 3 Dipartimento di Biologia, Università degli Studi di Padova, Padova, Italy; 4 Unit of Neurology 5 and Neuropathology, Fondazione IRCCS Istituto Neurologico Carlo Besta, Milan, Italy

**Keywords:** neurodegeneration, prion diseases, PrP^C^, PrP^Sc^, prions, tau, tauopathy, aggregation, tau fibrils, conformers

## Abstract

Neurodegenerative diseases are often characterized by the codeposition of different amyloidogenic proteins, normally defining distinct proteinopathies. An example is represented by prion diseases, where the classical deposition of the aberrant conformational isoform of the prion protein (PrP^Sc^) can be associated with tau insoluble species, which are usually involved in another class of diseases called tauopathies. How this copresence of amyloidogenic proteins can influence the progression of prion diseases is still a matter of debate. Recently, the cellular form of the prion protein, PrP^C^, has been investigated as a possible receptor of amyloidogenic proteins, since its binding activity with Aβ, tau, and α-synuclein has been reported, and it has been linked to several neurotoxic behaviors exerted by these proteins. We have previously shown that the treatment of chronically prion-infected cells with tau K18 fibrils reduced PrP^Sc^ levels. In this work, we further explored this mechanism by using another tau construct that includes the sequence that forms the core of Alzheimer’s disease tau filaments *in vivo* to obtain a distinct fibril type. Despite a difference of six amino acids, these two constructs form fibrils characterized by distinct biochemical and biological features. However, their effects on PrP^Sc^ reduction were comparable and probably based on the binding to PrP^C^ at the plasma membrane, inhibiting the pathological conversion event. Our results suggest PrP^C^ as receptor for different types of tau fibrils and point out a role of tau amyloid fibrils in preventing the pathological PrP^C^ to PrP^Sc^ conformational change.

Prion diseases are a heterogeneous group of fatal and infectious neurodegenerative pathologies caused by the structural conversion of the cellular prion protein (PrP^C^) into the disease-causing scrapie isoform (PrP^Sc^), which accumulates mainly extracellularly (1). Human prion diseases are clinically and pathologically diverse, with most of the cases classified as sporadic Creutzfeldt-Jakob disease (CJD), and the remaining ones as genetic, such as familial CJD, Gerstmann-Sträussler-Scheinker disease (GSS), and fatal familial insomnia, and acquired, representing less than 1% of all cases (2).

Several groups reported the presence of phosphorylated tau deposits in GSS patients, assuming neurofibrillary tangles morphology with paired helical filaments (PHFs) (3, 4, 5, 6). In 1997, Tranchant *et al.* reported the presence of neurofibrillary tangles in a GSS patient affected by the A117V mutation in the prion protein gene. Interestingly, tau deposition in this patient could be related to either the protracted duration of the disease or the patient’s age, as the clinical course was the longest in his family (7). Other works highlighted the colocalization between prion plaques and phosphorylated tau deposits in sporadic CJD and variant CJD cases (8, 9, 10).

Several lines of evidence suggest PrP^C^ as the mediator of the neurotoxic effects and the spreading of pathological aggregates that, besides PrP^Sc^ in prion diseases (11), are linked to other neurodegenerative disorders (12). PrP^C^ has been identified as one of the binding partners for synaptotoxic oligomers of amyloid β (Aβ) (13), mediating their cognitive detrimental effects (14). Anti-PrP antibodies preventing the interaction between PrP^C^ and Aβ efficiently hampered the Aβ-mediated disruption of synaptic plasticity (15). Similarly, pathological α-synuclein spreads faster in PrP^C^-overexpressing mice (16), thus suggesting an involvement of the cellular prion protein in α-synucleinopathies (17). Moreover, our group recently reported a PrP-dependent uptake and toxicity of TAR DNA-binding protein-43 (TDP-43) fibrils (18).

We have previously shown a similar interplay between PrP^C^ and tau amyloids (19). The N-terminal region of PrP^C^ was shown to interact with recombinant tau K18 fibrils and their uptake resulted less efficient in Neuro 2a (N2a) cells knocked out for PrP^C^. Moreover, tau K18 fibrils promoted the increased localization of PrP^C^ at the plasma membrane.

Interestingly, tau amyloid administration significantly reduced PrP^Sc^ level in N2a cells chronically infected with the RML prion strain, probably through an inhibitory effect on prion conversion. However, we were unable to exclude whether, besides PrP^C^ binding, tau fibrils could affect prion load by other mechanisms.

To further investigate the mechanism responsible for the tau-mediated prion reduction and to compare the effect of distinct conformers of tau fibrils on PrP^Sc^, we designed a new tau construct that, compared to K18, has a six amino acids longer sequence (amino acids 244–378).

## Results

### Tau amyloid fibrils reduce prions burden in a time-dependent manner

To better understand the reduction in prion load observed in our previous study (19), we employed, together with tau K18, a second tau construct, herein called tau 244–378. This fragment encompasses the sequence shown to be important for the packing interface of the core of Alzheimer’s disease (AD) tau PHFs and straight filaments (SFs) (20). The addition of the six C-terminal amino acids to tau K18 (amino acids 244–372) was performed using the restriction-free cloning technique, and the protein was expressed and purified as previously described for tau K18 (19) (Fig. S1*A*). To produce *in vitro* amyloid fibrils, both tau proteins were subjected to the same fibrillization protocol (19). As shown in Fig. S1*B*, both tau K18 and 244–378 showed comparable aggregation kinetics, even if K18 reached a higher thioflavin T (ThT) fluorescence signal compared to 244–378. The end-stage products of the fibrillization process were characterized by transmission electron microscopy (TEM) after sonication to break long fibrils into smaller species. TEM analysis showed that both constructs form fibrillar structures *in vitro* (Fig. S1*C*). To further characterize tau 244–378 fibrils, both fibrillization products were subjected to digestion with increasing concentrations of proteinase K (PK), followed by Western blot (WB) analysis, as it was already shown that the degree of resistance to proteases and the digestion pattern correlate with the structural organization of both *in vitro* and brain-isolated tau amyloids (21, 22, 23). As shown in Fig. S2*A*, both constructs form fibrils characterized by the resistance to PK digestion, highlighted by the presence of species with high molecular weight. In particular, tau K18 fibrils showed a higher resistance to PK digestion than tau 244-378. These latter were completely digested at concentrations higher than 1 μg/ml after 1 h of incubation at 37 °C (Fig. S2*A*). In contrast, tau K18 fibrils were not completely digested neither at the highest concentration of PK used (100 μg/ml) nor after increasing the time of the treatment up to 24 h (Fig. S2, *B* and *C*). These results suggest that the two tau constructs acquire distinct conformations when subjected to *in vitro* fibrillization. 3-(4,5-dimethylthiazol-2-yl)-2,5-diphenyltetrazolium bromide (MTT) assay in RML prion-infected N2a (ScN2a RML) cells showed that tau 244-378 fibrils caused a significant alteration in their metabolic activity, even at the lowest concentration (Fig. S3*A*), while tau K18 did not (19). However, as we did not observe major effects on cell proliferation and viability at 2 μM, also 244-378 fibrils were used in cell culture experiments (Fig. S3*B*).

**Figure 1:**
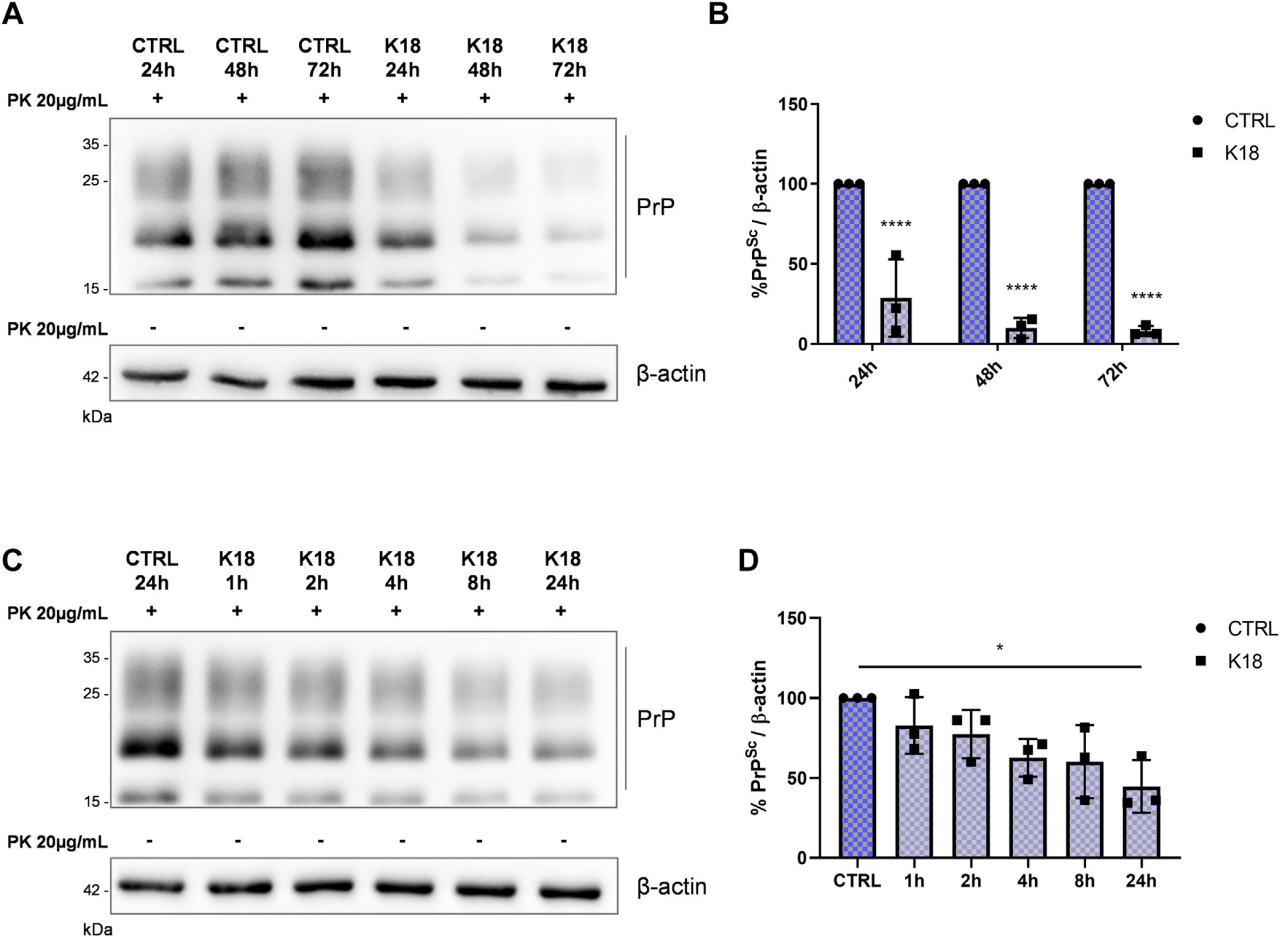
**Tau fibrils-mediated PrP**^**Sc**^**reduction.***A* and *B*, representative WB (*A*) and related quantification (*B*) of PrP^Sc^ in ScN2a RML cells following 2 μM tau K18 fibrils incubation for 24, 48, or 72 h, after which cells were kept in culture without the treatment up to 72 h. β-actin of the same samples not treated with PK was used as loading control. *B*, quantification of three independent experiments. Values are shown as percentage of PrP^Sc^ relative to β-actin. Data are reported as mean ± SD. Data were analyzed with two-way ANOVA with Sidak’s multiple comparisons test: ∗∗∗∗*p* ≤ 0.0001. *C* and *D*, representative WB (*C*) and related quantification (*D*) of PrP^Sc^ levels in ScN2a RML cells incubated with 2 μM of tau K18 fibrils for 1, 2, 4, 8, and 24 h and kept in culture without the treatment up to 72 h. β-actin of the same samples not treated with PK was used as loading control. *D*, quantification of three independent experiments. Values are shown as percentage of PrP^Sc^ relative to β-actin. Data are reported as mean ± SD and were analyzed with Friedman test with Dunn’s multiple comparisons test: ∗*p* ≤ 0.05. N2a, Neuro 2a; PK, proteinase K; PrP^Sc^, isoform of the prion protein; ScN2a RML, RML prion-infected N2a; WB, Western blot.

As previously shown, tau K18 fibril treatment caused a reduction in PrP^Sc^ levels after 72 h of continuous incubation (19). To evaluate whether PrP^Sc^ reduction needs 72 h of continuous incubation with tau fibrils or whether tau fibrils act earlier, we tested different tau K18 incubation times (72 h, 48 h, 24 h, 8 h, 4 h, 2h, and 1 h) before treatment removal and culturing up to 72 h. The PK-resistant PrP^Sc^ content was evaluated by WB. As shown in Figure 1, *A*–*D*, shorter incubation times with tau K18 fibrils caused a reduction in PrP^Sc^ levels at 72 h, suggesting that tau effect starts before the clearance of PrP^Sc^ appears.

**Figure 2:**
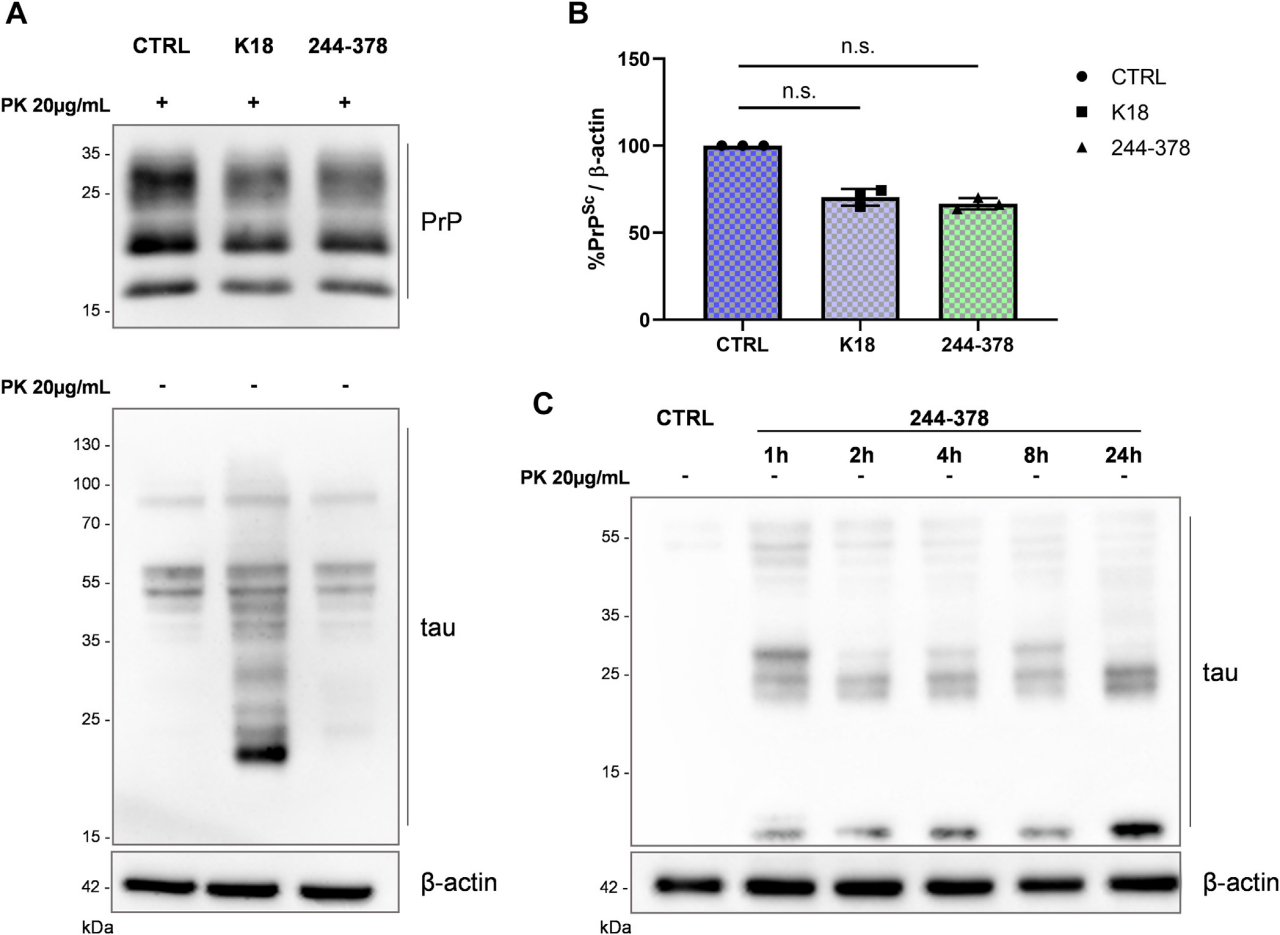
**Comparison of tau K18 and 244-378 fibrils on PrP**^**Sc**^**levels.***A*, representative WB of ScN2a RML cells after their exposure for 4 h to 2 μM of tau K18 and 244-378 fibrils followed by PBS 1X washes to remove the treatment and culturing up to 72 h to assess PrP^Sc^ reduction. PK digestion was performed to reveal PrP^Sc^ content (*upper panel*). The same samples not treated with PK and probed with anti-tau antibody were tested for the presence of intracellular tau K18 and 244-378 fibrils (*lower panel*). β-actin was used as loading control. *B*, quantification of the PrP^Sc^ level from three independent experiments. Values are shown as percentage of PrP^Sc^ relative to β-actin and data reported as mean ± SD. Data were analyzed with Friedman test with Dunn’s multiple comparisons test: n.s. not significant. *C*, internalization of tau 244-378 fibrils. WB analysis of ScN2a RML cells treated with 2 μM of tau 244-378 fibrils for 1, 2, 4, 8, and 24 h and then lysed and tested for the intracellular presence of tau 244-378 fibrils. β-actin was used as loading control. N2a, Neuro 2a; PK, proteinase K; PrP^Sc^, isoform of the prion protein; ScN2a RML, RML prion-infected N2a; WB, Western blot.

To investigate whether the treatment with tau 244-378 fibrils resulted in the same prion reduction, a comparison with K18 was also performed. For this experiment, we chose 4 h of incubation, since it was the lowest K18 incubation time showing around 50% of PrP^Sc^ reduction. As shown in Figure 2, *A* (upper panel) and *B*, both tau species were able to reduce PrP^Sc^ level. We also checked whether tau amyloids were actively internalized by cells, as they could act on intracellular degradation mechanisms to promote PrP^Sc^ clearance. Therefore, we evaluated in the same samples the presence of intracellular tau amyloids at 72 h. Extracellular tau fibrils were removed by trypsinization to evaluate the signal coming exclusively from intracellular fibrils (24, 25, 26, 27). Interestingly, following 4 h of incubation, cells treated with K18 fibrils showed a strong tau signal corresponding to the intracellular aggregates which are still present after 72 h of culturing, while a faint tau signal was detected in cells treated with 244-378 fibrils (Fig. 2*A* lower panel). However, the cells treated with tau 244-378 fibrils, checked right after 1, 2, 4, 8, and 24 h of incubation, showed higher amounts of internalized aggregates (Fig. 2*C*), demonstrating that both fibril types are actively internalized by cells.

### Tau fibrils are internalized and localize in the autophagolysosomal pathway

To further characterize the PrP^Sc^ clearance mediated by tau fibrils, we assessed their localization following the uptake in ScN2a cells, as it may underlie specific pathways involved in the degradation of both tau fibrils and prions. Tau K18 fibrils were conjugated to Alexa-488 dye to produce fluorescent fibrils that were administered to cells for 24 h to obtain a significant internalization. Cells were then stained with antibodies targeting several cell compartments. Trypan blue was used before fixation to quench the signal of fibrils that were not internalized, as this dye cannot enter living cells (28). Images of a single cell were captured as multiple sections along the z-axis to take into account the whole cell volume and shown in two-dimensional with orthogonal planes, to assess the colocalization both in the XZ and YZ axes. As shown in Fig. S4, tau K18 fibrils are efficiently internalized after 24 h of incubation, localizing in subcellular compartments such as Golgi apparatus (M6PR), autophagosomes (LC3B), and lysosomes (LAMP2). No colocalization was observed with the early endosome marker EEA1 and the endoplasmic reticulum one (Calnexin).

To investigate the potential role of the lysosomal pathway in mediating prion clearance induced by tau K18 fibrils, we took advantage of different inhibitors known to block the autophagolysosomal pathway at different steps. Despite the efficient inhibition of the autophagolysosomal pathway, as shown by the levels of LC3B proteins, neither 3-methyladenine, bafilomycin A1, nor chloroquine were able to impair the clearance of PrP^Sc^ mediated by tau K18 fibrils (Fig. S5, *A*–*C*). At the same time, the treatment with MG132, known to inhibit proteasomal degradation, had no effect (Fig. S5*D*), suggesting that the most common degradation pathways, normally implicated in the removal of misfolded proteins, are not involved in the tau amyloid fibrils-mediated PrP^Sc^ reduction.

### Tau fibril uptake is not required for PrP^Sc^ reduction

**Figure 3:**
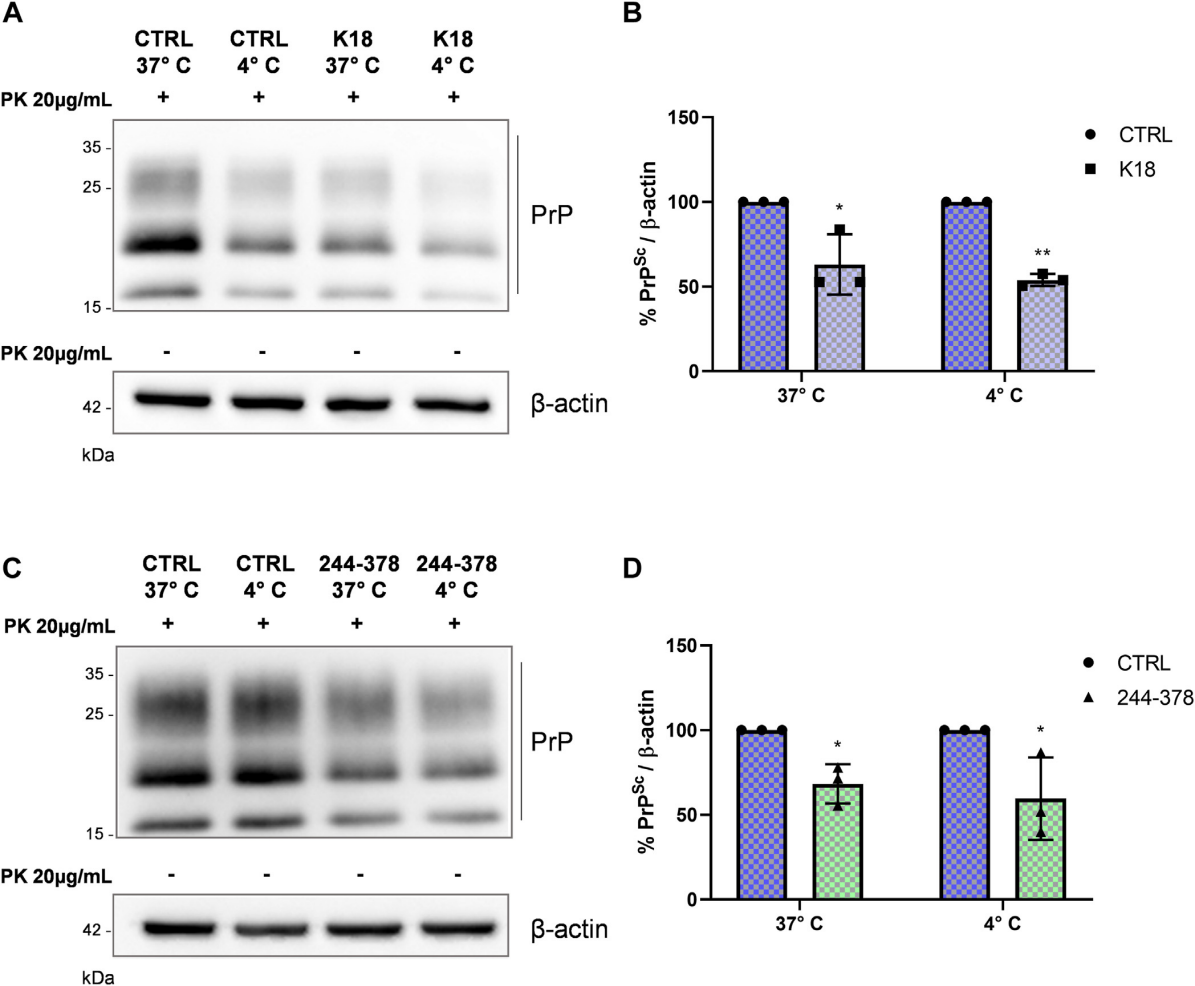
**Effect of the inhibition of tau fibril uptake on PrP**^**Sc**^**.***A* and *B*, representative WB (*A*) and related quantification (*B*) of ScN2a RML cells incubated for 4 h with 2 μM of tau K18 fibrils at 37 °C and 4 °C and, following PBS 1× washes to remove the treatment, kept in culture up to 72 h. β-actin of the same samples not treated with PK (Fig. S6*B*) was used as loading control. *B*, graph shows the quantification of three independent experiments. Values are shown as percentage of PrP^Sc^ relative to β-actin and data reported as mean ± SD. Data were analyzed with two-way ANOVA with Sidak’s multiple comparisons test: ∗*p* ≤ 0.05, ∗∗*p* ≤ 0.01. *C* and *D*, representative WB and related quantification (*D*) of ScN2a RML cells incubated for 4 h with tau 244-378 fibrils at 37 °C and 4 °C and, following PBS 1X washes to remove the treatment, kept in culture up to 72 h. β-actin of the same samples not treated with PK (Fig. S6*C left* lanes) was used as loading control. *D*, graph shows the quantification of three independent experiments. Values are shown as percentage of PrP^Sc^ relative to β-actin and data reported as mean ± SD. Data were analyzed with two-way ANOVA with Sidak’s multiple comparisons test: ∗*p* ≤ 0.05. N2a, Neuro 2a; PK, proteinase K; PrP^Sc^, isoform of the prion protein; ScN2a RML, RML prion-infected N2a; WB, Western blot.

We have shown that tau fibrils efficiently reduce prion level in ScN2a RML cells, and they are internalized by cells, localizing in different subcellular compartments. However, if internalization is involved, there should be other mechanisms by which tau fibrils promote PrP^Sc^ reduction, since we have already ruled out the contribution of cellular degradation pathways. To assess whether tau fibril internalization is somehow related to PrP^Sc^ reduction, we incubated ScN2a RML cells at 4 °C. At this restrictive temperature, all the energy-dependent endocytic processes are inhibited, but tau amyloid fibrils are still able to bind to the plasma membrane (24). As already shown for the uninfected N2a cells (19), also for ScN2a RML, the incubation at 4 °C almost completely inhibited the uptake of Alexa488-labeled tau K18 fibrils (Fig. S6*A*). We then evaluated the PrP^Sc^ reduction at 72 h, after 4 h of incubation with tau K18 fibrils both at 37 °C and 4 °C. As shown in Fig. S6*B*, WB analysis of intracellular tau K18 fibrils at 4 °C revealed an almost complete absence of signal when compared to 37 °C, confirming the blockage of tau fibril endocytosis and the validity of our experimental design. However, no major differences were observed in PrP^Sc^ clearance, with a similar decrease in K18-treated cells either at 37 °C or 4 °C (Fig. 3, *A* and *B*). This result suggests that despite their internalization, tau K18 fibrils do not mediate PrP^Sc^ reduction by entering the cells.

We then assessed whether tau 244-378 fibrils, characterized by different biochemical and biological behaviors compared to K18, may act on PrP^Sc^ reduction with similar mechanisms. As previously shown, tau 244-378 fibrils are less resistant to protease degradation compared to K18 fibrils, and despite being quickly internalized, they are efficiently removed after 3 days of incubation. To overcome this problem, the blocking of tau 244-378 fibril internalization at 4 °C was performed by incubating cells for 4 h followed by cell lysis to prevent cells from eliminating fibrils. Fig. S6*C* (right lanes) shows how, as for tau K18 fibrils, 244-378 fibril internalization is efficiently reduced by incubating cells at 4 °C. Moreover, the complete absence of tau signal when, after the treatment, cells are left in incubation up to 72 h shows that after the initial uptake, 244-378 fibrils are efficiently removed by cells, probably degraded (Fig. S6*C*, left lanes). However, after 3 days, the 244-378-mediated reduction in PrP^Sc^ levels between cells incubated at 37 °C and 4 °C was comparable (Fig. 3, *C* and *D*).

These results show that despite being internalized and exhibiting different biochemical and biological properties, both K18 and 244-378 fibrils reduce prion level independently of their uptake.

### Tau amyloid fibrils bind to PrP^C^ and hinder PrP^Sc^*de novo* infection

Since the effect of tau fibrils on prion clearance is independent from their internalization, their action could be exerted in the extracellular environment. Being mainly located on the outer leaflet of the plasma membrane, PrP^C^ may represent the target of tau amyloid fibrils used in this study. As previously shown, the administration of tau K18 fibrils to N2a cells caused an increased localization of PrP^C^ in the plasma membrane, probably due to its impaired recycling and its stabilization in this compartment (19). To confirm the binding observed between PrP^C^ and tau amyloid fibrils (19, 29), we performed an enzyme-linked immunosorbent assay (ELISA) experiment using both tau fibril types. As shown in Fig. S7, both tau K18 and 244-378 fibrils bind to the recombinant mouse PrP_23-231_ with an average EC_50_ of 102.8 and 174.47 nM respectively, whereas no appreciable affinity was detected with the bovine serum albumin used as negative control. This result confirms the ability of PrP^C^ to bind not only amyloid aggregates of different proteins (29) but also distinct fibril types of tau, suggesting that the reduction in PrP^Sc^ level, observed so far in our cellular model, could derive from a direct interaction between tau amyloids and PrP^C^, making the latter unavailable for the conversion into PrP^Sc^.

To reproduce the effect of tau fibrils on PrP^Sc^-mediated PrP^C^ conversion, we took advantage of an *in vitro* real-time quaking-induced conversion (RT-QuIC) assay. Recombinant mouse PrP_23-231_ (rPrP) was preincubated with tau K18 or 244-378 fibrils in 5:1 and 1:1 M ratios, before seeding with 1 ng of phosphotungstate precipitated PrP^Sc^. ThT fluorescent dye was added to monitor the real-time aggregation process. As shown in Fig. S8, both tau K18 (upper panel) and 244-378 (lower panel) fibrils hinder the PrP^Sc^-mediated rPrP conversion in a molar concentration-dependent manner.

However, besides the binding to PrP^C^, a direct interaction between tau fibrils and PrP^Sc^ cannot be ruled out.

Regardless of the binding to either PrP^C^ or PrP^Sc^, tau fibril administration should be able to hinder the conversion of PrP^C^ also in uninfected cells.

**Figure 4:**
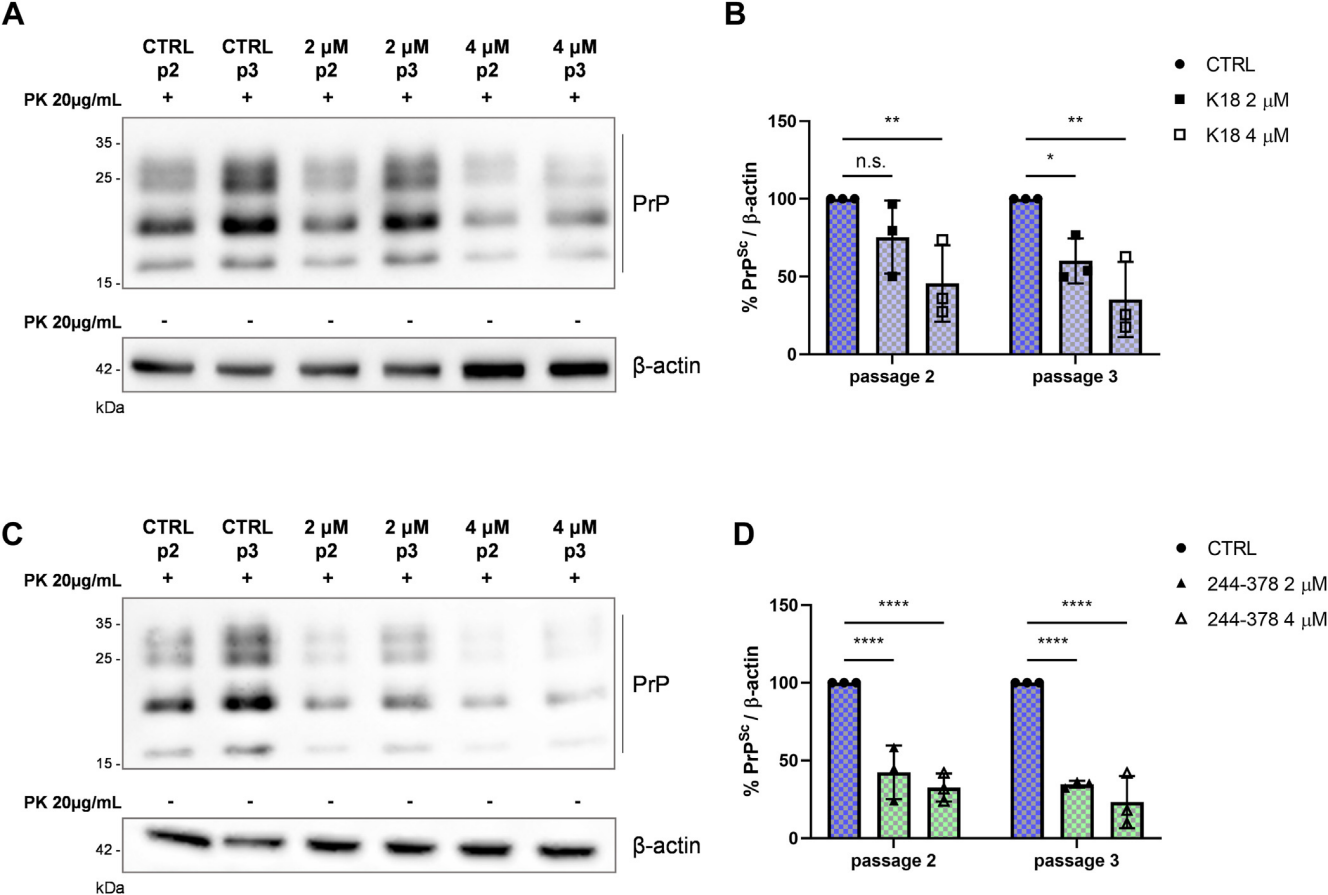
**Tau fibril effect on *de novo* infection.***A* and *B*, representative WB (*A*) and related quantification (*B*) of N2a cells pretreated for 4 h with 2 or 4 μM of tau K18 fibrils and then infected with ScN2a RML homogenate. Cells were passaged for three times to allow prion replication. β-actin of the same samples not treated with PK was used as loading control. *B*, graph shows the quantification of three independent experiments. Values are shown as percentage of PrP^Sc^ relative to β-actin and data reported as mean ± SD. Data were analyzed with two-way ANOVA with Sidak’s multiple comparisons test: n.s. not significant, ∗*p* ≤ 0.05, ∗∗*p* ≤ 0.01. *C* and *D*, representative WB (*C*) and related quantification (*D*) of N2a cells pretreated for 4 h with 2 or 4 μM of tau 244-378 fibrils and then infected with ScN2a RML homogenate. Cells were passaged for three times to allow prion replication. β-actin of the same samples not treated with PK was used as loading control. *D*, graph shows the quantification of three independent experiments. Values are shown as percentage of PrP^Sc^ relative to β-actin and data reported as mean ± SD. Data were analyzed with two-way ANOVA with Sidak’s multiple comparisons test: ∗∗∗∗*p* ≤ 0.0001. N2a, Neuro 2a; PK, proteinase K; PrP^Sc^, isoform of the prion protein; ScN2a RML, RML prion-infected N2a; WB, Western blot.

To test this hypothesis, we took advantage of a *de novo* prion infection protocol, which allows PrP^C^ to PrP^Sc^ conversion in uninfected cells using an inoculum obtained from prion-infected cells (30). To assess the contribution of tau fibrils in preventing the infection, N2a cells were pretreated for 4 h with 2 or 4 μM of tau K18 fibrils, washed with PBS to remove the treatment, and then exposed for 72 h to ScN2a RML cell lysate. The efficient *de novo* cell infection is shown by the increase of PrP^Sc^ content from passage 2 to passage 3, suggesting that cells are actively replicating prions (Fig. 4, *A* and *C*).

Tau K18 fibril pretreatment, either at 2 μM and 4 μM, was efficiently able to reduce prion accumulation in both passages, confirming the impairment of the *de novo* infection process (Fig. 4, *A* and *B*).

As expected, a similar result was obtained performing the *de novo* infection experiment with tau 244-378 fibrils (Fig. 4, *C* and *D*).

## Discussion

Although Aβ, tau, α-synuclein, prion, and TDP-43 aggregates are usually involved in different pathologies, their accumulation often occurs in nondiseased individuals, and they can codeposit in the brain of diseased patients (31). The same also applies to prion diseases, with described cases of codeposition of PrP^Sc^ with Aβ (10), α-synuclein (32) and phosphorylated tau (3, 4, 5, 6, 7, 8, 9, 10). On this regard, it is now widely recognized that the cellular form of the prion protein, PrP^C^, binds different β-sheet-enriched amyloid proteins, suggesting its affinity to a common feature of these amyloids. Recently, Corbett *et al.* showed that PrP^C^ binds to soluble aggregates of tau, α-synuclein, and Aβ and that its genetic ablation reduced the toxic effects that these aggregates normally exert (29). Similarly, our lab has shown an interplay between PrP^C^ and α-synuclein amyloid fibrils, with an increased uptake and spreading of α-synuclein aggregates in the presence of PrP^C^, both *in vitro* and *in vivo* (17). Moreover, the N terminus of PrP^C^ was found to interact with tau K18 amyloid fibrils, facilitating their uptake in N2a cell line (19). Interestingly, α-synuclein, tau, and TDP-43 fibrils were able to reduce the amount of PrP^Sc^ when administered to prion-infected N2a cells, possibly by hindering the conversion event of PrP^C^. To further explore this effect, we took advantage of the previously used tau K18 construct, made of the amino acids 244-372, and of a new tau construct, which extends the tau K18 sequence of six amino acids (244–378). This fragment encompasses the sequence shown to be important for the packing interface of the core of AD tau PHFs and SFs, and it could result in the formation of filaments with a different structure compared to those of K18. TEM analysis of the end-stage products revealed that as for tau K18, also 244-378 forms fibrils *in vitro*. We further characterized 244-378 fibrils by subjecting them to protease degradation, as the resistance to proteolysis represents one of the features of *in vitro* and brain-extracted amyloid proteins (21, 22, 23, 33), and it correlates with the structural organization and the amount of β-sheets (34). Surprisingly, we found that 244-378 fibrils, unlike the K18 ones, were very sensitive to PK digestion, suggesting different conformational structures and different β-sheet content, that may explain also the lower ThT fluorescence emission when fibrillized *in vitro*. This result may also suggest that 244-378, comprising the *in vivo* PHFs and SFs AD sequence, may reflect a more physiological conformational structure, as it was already shown that *in vivo* filaments are less resistant to protease degradation when compared to the *in vitro* produced ones (23, 35). First, we tested the metabolic effect of 244-378 fibrils in our cellular model. In contrast to tau K18 fibrils, tau 244-378 fibrils exhibited a marked effect in the reduction of the metabolic activity in ScN2a RML cells, further confirming not only the biochemical but also the different biological nature of these two amyloid preparations. However, at 2 μM concentration, no effects on cell proliferation and viability were observed.

As previously shown, tau K18 fibrils reduce PrP^Sc^ level after 72 h of incubation (19). Here, we have shown that both tau K18 and 244-378 fibrils were spontaneously internalized by these cells and caused a comparable reduction effect on prion load, despite the 244-378 signal was almost absent after 72 h, probably because of their degradation. Their active digestion by cells would be in agreement with the lower resistance of 244-378 fibrils to protease degradation, and it may also explain the reduced metabolic activity observed in the MTT assay. We also observed that, following the internalization, tau K18 fibrils localized in the Golgi apparatus and the autophagolysosomal pathway, suggesting a possible stimulation of the cellular degradation pathways and a consequent decrease in PrP^Sc^ levels. However, the treatment with inhibitors, aimed at blocking the autophagic and proteasomal degradation, had no effects, thus ruling out a role of these pathways in the tau fibrils–mediated PrP^Sc^ clearance. Unexpectedly, we observed that tau fibril internalization did not affect their ability to reduce PrP^Sc^, as prion reduction was fully comparable between cells incubated at 37 °C or 4 °C. These findings strongly suggest that tau fibrils–dependent PrP^Sc^ clearance is mediated by a direct binding between PrP^C^/PrP^Sc^ and tau fibrils at the plasma membrane, hindering one of the two partners necessary for prion conversion. We already observed the increased PrP^C^ clustering at the plasma membrane following tau fibril exposure (19). By exploiting their action on the plasma membrane, the binding of tau fibrils with PrP^Sc^ seems unlikely, since the latter is mostly present at the intracellular level (36, 37) with little localization at the plasma membrane (38). We demonstrated here that both tau K18 and 244-378 fibrils efficiently bind to the recombinant full-length PrP, with EC_50_ values slightly higher than the ones found for other amyloid species (29), confirming the ability of the cellular prion protein to act as a receptor not only for different amyloidogenic proteins but also for distinct types of tau fibrils. This result may suggest that the interaction between tau fibrils and PrP^C^ reduces PrP^Sc^ levels by sequestering one of the two partners needed for the infectious cycle. Indeed, both tau amyloids efficiently hindered the PrP^Sc^-mediated rPrP conversion in an *in vitro* RT-QuIC assay, thus confirming their interference in the prion infection process. After the observation of tau fibrils-mediated reduction of PrP^Sc^ level in chronically prion-infected cells, we wondered whether this effect is limited to an established prion infection or whether tau amyloids can somehow affect the early stages of prion infection. To test this hypothesis, we took advantage of a *de novo* prion infection protocol, by exposing uninfected N2a cells to a homogenate of ScN2a cells. In this way, the PrP^Sc^ present in the inoculum converts the PrP^C^ of the uninfected N2a cells. After the initial infection step, cells were passaged three times to allow the removal of the initial inoculum and the onset of the *de novo* prions. The first passage was not analyzed to avoid the interference of the initial inoculum. Our results indicated that both tau K18 and 244-378 fibrils, administered before the exposure of cells to prion homogenate, reduced significantly the accumulation of PrP^Sc^ following the *de novo* infection.

In addition, our *de novo* experiment suggests that, since the treatment is removed before the addition of PrP^Sc^ inoculum, tau fibril binding to PrP^C^ represents the event through which they exert their effect on PrP^Sc^. In the early phases of the infection process, tau binding to PrP^C^ could prevent its physical interaction with PrP^Sc^, inhibiting the conversion, since the addition of tau fibrils is sufficient to hinder this process *in vitro*. Furthermore, tau fibril binding to PrP^C^ could costrain the protein at the plasma membrane, as demonstrated by its clustering in this compartment following tau fibril exposure (19). This could result in the hampering of PrP^C^ recycling through the endocytic compartments preventing its interaction with PrP^Sc^ and the progress of the infectious cycle. During the established infection, tau fibril binding to PrP^C^ may stabilize the protein at the plasma membrane, preventing its interaction with PrP^Sc^ in the endocytic compartments, where the conversion most likely occurs (36, 39, 40).

In conclusion, our findings confirmed the role of tau fibrils in mediating the reduction of prions in ScN2a RML cells. Moreover, this effect seems to reside in the ability of tau fibrils to bind to the PrP^C^ present on the plasma membrane, thus stabilizing the protein and hindering its conversion to PrP^Sc^. Here we show that PrP^C^ may act as a receptor for different tau fibril types, that, despite showing different conformational properties, are able to comparably reduce prions in our chronically prion-infected model. In addition, tau amyloid fibrils impair the initial phases of prion conversion, hindering PrP^C^/PrP^Sc^ interaction and/or stabilizing PrP^C^ at the plasma membrane. These results may suggest a dual interplay between prion protein and other amyloidogenic proteins. While, as previously shown for tau, α-synuclein, and TDP-43, the presence of PrP^C^ at the plasma membrane facilitates the uptake of pathological aggregates possibly accelerating the course of the disease, here we show that tau circulating species may protect PrP^C^ from PrP^Sc^ interaction, thus slowing prion conversion and prion disease progression. This may also explain why in some prion-affected patients, the presence of tau aggregates may correlate with a longer disease incubation. In this regard, understanding the reciprocal interactions between different amyloidogenic proteins and how these relationships may result in distinct disease phenotypes could represent a key step in the development of targeted therapies.

## Experimental procedures

### Tau production and fibrillization

Human tau 244-378 construct was obtained using the restriction-free cloning technique. Forward (GGGAGGCGGCAACAAAAAAATTGAGACCCACAAGCTGACCTTCT) and reverse (GCTTTGTTAGCAGCCGGATCCTTATCAGAAGGTCAGCTTGTGGGT) primers were designed to add six amino acids to the C terminus of tau K18 (amino acids 244–372 of the human tau sequence) in a pET-11a plasmid.

Expression and purification of human tau proteins were performed as previously described (41). Briefly, pET-11a plasmids, containing the sequences encoding for the human tau constructs, were transformed in *Escherichia coli BL21 DE3*. Cells were grown in 2 L of Luria-Bertani medium supplemented with 100 μg/ml, and the expression of the protein was obtained with 0.8 mM isopropyl β-D-1-thiogalactopyranoside (PanReac AppliChem). Proteins were purified by a first step of cation-exchange chromatography (HiTrap SP-FF, Cytiva) and a second one of size-exclusion chromatography (HiLoad 26-600, Cytiva). Purified proteins were analyzed in sodium dodecyl sulphate-polyacrylamide gel electrophoresis (SDS-PAGE), lyophilized, and stored at −80 °C.

Fibrillization reactions were performed in a 96-well black plate with transparent bottom (BD Falcon) with a 3 mm glass bead (Sigma-Aldrich). The 200 μl final volume mixtures were composed of tau K18/244-378 0.5 mg/ml, 0.1 mM DTT, and 50 μg/ml heparin in PBS 1X pH 7.4. Due to ThT cytotoxic effect, 10 μM of the dye was added only in three wells to monitor the real-time aggregation. Plates were covered with a sealing tape (Fisher Scientific) and incubated at 37 °C in orbital shaking (50 s of 400 rpm shaking and 10 s of rest) in FLUOstar Omega Microplate Reader (BMG LABTECH). Fluorescence was monitored every 30 min by bottom reading at 444 nm of excitation and 485 nm of emission. The reaction was stopped after 40 h, and fibrils were pelleted by centrifugation at 186.000*g* for 1 h at 4 °C and resuspended in 1 ml of 1× PBS pH 7.4 before being stored at −80 °C. Before use, aliquots were sonicated for 5 min in a sonicator Misonix s3000 at 250 W.

### Recombinant PrP production

Mouse PrP_23-231_ construct was transformed in *E. coli* BL21 (DE3) cells (Stratagene), and its expression was induced with 1 mM isopropyl β-D-1-thiogalactopyranoside (PanReac AppliChem). Cells were grown at 30 °C for 12 h and then lysed using PandaPLUS 2000. Inclusion bodies containing the recombinant protein were washed several times in bi-distilled water and then dissolved in 8 M guanidine hydrochloride. Protein was purified by size-exclusion chromatography into a pre-equilibrated HiLoad 26/60 Superdex 200 pg column (Cytiva). Protein refolding was performed by dialysis against 20 mM sodium acetate, pH 5.5. The purified protein was analyzed by SDS-PAGE under reducing conditions, dialyzed against PBS 1X, pH 5.8, and stored at −80 °C. All salts used were from Sigma-Aldrich.

### TEM analysis

Ten microliter of tau K18 or 244-378 fibril solution was dropped onto 200-mesh Formvar carbon–coated nickel grids (Electron Microscopy Sciences) for 20 min after which samples were stained with 25% uranyl acetate replacement (Electron Microscopy Sciences) for 10 min. Before the analysis, the staining solution was removed using Whatman filter paper, and the grids were air-dried for 5 min. Samples were visualized using a FEI Tecnai Spirit Transmission Electron Microscope operating at 120 kV and equipped with an Olympus MegaView G2 camera.

### Cell cultures

Mouse neuroblastoma N2a cells were kindly provided by Prof Chiara Zurzolo (Unité de traffic membranaire et pathogenèse, Institute Pasteur). ScN2a cells are clones persistently infected with the RML prion strain as described by Prusiner's group (42). Cells were kept in culture at 37 °C and 5% CO_2_ in humified atmosphere with minimal essential medium + GlutaMAX (Thermo Fisher Scientific) supplemented with 10% fetal bovine serum (Euroclone), 1X non-essential amino acids (Euroclone) and 1× penicillin–streptomycin solution (Euroclone) and split every 3 to 5 days using Trypsin-EDTA 1X solution (Sigma-Aldrich).

### Cell treatments

ScN2a RML or N2a were plated in 6 cm or 10 cm Petri dishes according to the experimental setting. Recombinant preformed fibrils at specific concentrations were directly administered to the medium of cultured cells and incubated for a different amount of time. In some experiments, non-internalized fibrils were removed by trypsinization after washing cells with PBS 1X.

For immunofluorescence experiments, tau K18 fibrils were conjugated to Alexa-488 succinimidyl ester (Thermo Fisher) according to the manufacturer’s instructions, and unbound dye was removed by subsequent dialysis (19).

To test the contribution of cellular degradation pathways, ScN2a RML cells were incubated for 72 h with 2 μM tau K18 fibrils and 10 mM 3-methyladenine (Sigma-Aldrich), and 100 mM bafilomycin A1 (Sigma-Aldrich) or 50 μM Chloroquine were added 16 h before lysis. For the inhibition of proteasomal activity, 5 μM of MG132 (Sigma-Aldrich) were added 16 h before lysis.

To block tau fibril endocytosis, ScN2a RML cells were preincubated for 10 min at 37 °C or 4 °C, after which 2 μM of tau fibrils were directly added to the medium, and cells were kept in culture for 4 h at 37 °C or 4 °C. Then, cells were washed twice with 1X PBS to remove the treatment and kept in culture up to 72 h at 37 °C.

### Analysis of internalized tau amyloids

For the evaluation of Alexa488-tau K18 fibril internalization, after incubation, cells were washed twice with sterile PBS 1X and incubated for 5 min with a sterile 1:1 solution of trypan blue in PBS. Trypan blue quenches the fluorescence coming from extracellular tau fibrils (28). Cells were then rinsed three times in PBS 1X and fixed with 4% paraformaldehyde for 30 min. Permeabilization was performed for 5 min in 0.1% Triton X-100 in PBS and coverslips stained with HCS CellMask Blue Stain (Thermo Fisher Scientific) 1:2500 labeling whole-cell cytoplasm. Coverslips were mounted with Fluoromount-G (Thermo Fischer Scientific) and stored at 4 °C. Images were acquired using a Nikon confocal microscope (Nikon A1plus).

### Metabolic cell activity and cell counts

The metabolic influence of tau 244-378 amyloid fibrils was evaluated with MTT assay. 10.000/well ScN2a cells were plated in a 96-well, and the day after, different concentrations of sonicated tau 244-378 fibrils were directly added to the medium, and cells were incubated for 72 h. Cells were then incubated for 3 h at 37 °C with 20 μl of 5 mg/ml MTT (Sigma-Aldrich) solution in PBS 1× followed by the solubilization with 1:1 DMSO/2-Propanol solution. The absorbance was measured at 570 nm in Enspire multimode plate reader (PerkinElmer) with a reference wavelength of 650 nm. Each condition was tested in six replicates and in three independent experiments.

Tau 244-378 fibril effects on proliferation and cell death were assessed by cell counting. 15.000/well ScN2a RML cells were plated in a 12-well, and the day after, they were treated with 2 μM tau 244-378 fibrils. Cells were left in incubation up to 72 h, after which they were detached with trypsin and counted using Scepter 2.0 Handheld Automated Cell Counter (Millipore) with 60 μM sensors. Three independent experiments were conducted, each one in three technical replicates.

### *De novo* prion infection

*De novo* prion infection of N2a cells was performed as previously described by Arshad *et al.* (30) with some modifications. Briefly, 150.000 N2a cells were plated in 12-wells, and the day after, pretreated with 2 or 4 μM tau K18/244-378 amyloids for 4 h and washed twice with PBS 1X to remove the treatment before the addition of the seed. PrP^Sc^ seed was prepared scraping a 10 cm Petri dish of ScN2a RML and sonicating cells in ice 2 × 5 s at 70% amplitude (Sonics VCX 130 PB). Total protein content of the lysate was quantified using bicinchoninic acid assay (Sigma-Aldrich), and 100 μg of proteins were added to N2a in a final volume of 500 μl of completed minimal essential medium without penicillin–streptomycin. Cells were kept in incubation with the seed for 72 h after which cells were detached with trypsin and plated 1:2 in a 6 cm Petri dish. Cells were split two more times every 4 days at 1:5 dilution, and the rest was pelleted and resuspended in 50 μl of lysis buffer and analyzed in WB for PrP^Sc^ presence after PK digestion. In all passages, penicillin–streptomycin solution was omitted. PrP^Sc^ amount from passage one was not analyzed to avoid the signal deriving from the initial seed.

### Proteinase K digestion

To detect PrP^Sc^ content, 150 μg of total proteins were digested with 20 μg/ml of PK (Sigma-Aldrich) at 37 °C for 1 h. The reaction was stopped by adding 2 mM of phenylmethylsulphonyl fluoride (Sigma-Aldrich), and samples were ultracentrifuged for 1 h at 186.000*g* at 4 °C. The resulting pellet was resuspended in 1× loading buffer and boiled for 10 min.

To assess fibril resistance to PK digestion, 2 μg of K18 or 244-378 fibrils were digested with 0.1, 1, 10, and 100 μg/ml of PK for 1 h at 37 °C.

2 μg of tau K18 fibrils were also digested with 100 μg/ml of PK for 1, 2, 4, 8, and 24 h at 37 °C.

### Western blotting

N2a and ScN2a were rinsed in PBS 1X and resuspended in lysis buffer (10 mM Tris HCl, 150 mM NaCl, 0.5% NP-40, 0.5% sodium deoxycholate). Lysates were centrifuged for 5 min at 5900*g* at 4 °C. Total protein content of N2a and ScN2a cells was quantified using bicinchoninic acid assay (Sigma-Aldrich). Proteins (20 μg) were diluted in loading buffer and samples boiled for 10 min at 100 °C.

Samples were loaded in 12/15% Tris-Glycine SDS-PAGE gels and transferred onto Immobilon P PVDF membranes (Millipore) for 2 h at 4 °C. Membranes were blocked in 5% nonfat milk in TBST and incubated overnight at 4 °C with mouse anti-PrP W226 1:1000 (kindly provided by Prof Robert Kammerer) (43), mouse anti-tau 7.51 1:500 (44), and rabbit anti-LC3B 1:1000 (2775, Cell Signaling) antibodies. After three washes in TBS-T, membranes were incubated with goat-anti-mouse IgG horse radish peroxidase (HRP)-conjugated antibody (Dako) for 1 h at room temperature (RT) and developed using Immobilon Classico Western HRP substrate (Millipore). Mouse anti β-actin-HRP antibody 1:10.000 (A3854, Sigma-Aldrich) was incubated for 1 h at RT and used for normalization. Images were acquired using Uvitec Alliance (Cambridge), and Uviband software was used for densitometric analysis.

### Immunofluorescence

For immunofluorescence experiments, 30.000 ScN2a RML cells were plated in 12-wells plate with a 12 mm coverslip coated for 1 h with Poly-L-lysine hydrobromide (Sigma-Aldrich) 100 μg/ml. The day after, cells were treated with 2 μM Alexa-488 tau K18 fibrils and incubated for 24 h. Cells were then rinsed in PBS 1X twice and fixed with 4% paraformaldehyde in PBS for 30 min. After permeabilization with 0.1% Triton X-100, coverslips were incubated for 1 h with blocking buffer (7% normal goat serum, 1% bovine serum albumin, 0.1% Triton X-100 in PBS) and then for 1 h and 30 min with the following primary antibodies in blocking buffer (rabbit anti-calnexin ab10286 1:500 Abcam, mouse anti-M6PR ab2733 1:500 Abcam, rabbit anti-EEA1 ab2900 1:500 Abcam, rat anti-LAMP2 ab25339 1:100 Abcam, and rabbit anti-LC3B 2775s 1:200 Cell Signaling). After PBS 1X washes, coverslips were incubated with the appropriate secondary Alexa594-conjugated antibody (Invitrogen) for 1 h and nuclei counter-stained with DAPI before mounting with Fluoromount-G (Thermo Fisher Scientific). Images were acquired with Nikon confocal (Nikon A1plus) as series of z-stacks, 0.25 μM step, 512 × 512. Plan Apo λ 60x Oil objective with a numerical aperture of 1.4 was used.

### ELISA assay

The binding between tau fibrils and the recombinant prion protein was assessed by ELISA. 96-well plates were coated overnight at 4 °C with 500 ng of K18 or 244-378 tau fibrils. Plates were washed three times with PBS + 0.05% Tween-20 (PBS-T) and blocked with 5% bovine serum albumin in PBS for 1 h at RT before the addition of different concentrations of recombinant mouse PrP_23-231_. After three subsequent washes with PBS-T, mouse anti-PrP W226 1 μg/ml was incubated for 1 h at RT, and then plates were incubated with goat-anti-mouse IgG horse-HRP–conjugated antibody (Dako) for 1 h. Plates were developed with 50 μl of 3,3′,5,5′-tetramethylbenzidine (Sigma-Aldrich), stopped by the addition of 1 M H_2_SO_4_ and read at 450 nm and 570 nm in Enspire multimode plate reader (PerkinElmer).

### RT-QuIC

To test the effect of tau fibrils on PrP conversion, recombinant PrP_23-231_ was preincubated for 30 min at RT with both tau K18 and 244-378 fibrils at 1:1 and 5:1 M concentration. The reaction mixture was then completed with 130 mM NaCl, 1 mM EDTA, 0.002% SDS, and 10 μM ThT in PBS 1X pH 7.4. All solutions were filtered with a 0.22 μM filter before use. In the seeded conditions, PrP^Sc^ was obtained by PTA precipitation (45), after which, 1 ng of seed was added. A 3 mm glass bead was added in each well. After sealing, the plate was incubated in FLUOstar Omega Microplate Reader (BMG LABTECH) at 37 °C, with cycles of 60 s shaking at 600 rpm and 60 s rest. ThT was used to monitor the real-time aggregation, and the fluorescence intensity was measured every 30 min by using 450 ± 10 nm (excitation) and 480 ± 10 nm (emission) wavelengths.

### Statistical analysis

Statistical analysis was performed using GraphPad Prism 8.0 software. Values were expressed as mean ± standard deviation (SD). Values are shown as percentage of PrP^Sc^/β-actin and compared to control group. Groups were analyzed with Friedman test (Dunn’s multiple comparisons test) and two-way ANOVA (Sidak’s multiple comparisons test). *p*-values ≤ 0.05 were considered statistically significant.

## Data availability

All data generated or analyzed during this study are included in this article (and its supporting information file).

## Supporting information

This article contains supporting information.

## Conflict of interest

The authors declare that they have no conflicts of interest with the contents of this article.
