# Supplementary material for: Different tau fibril types reduce prion level in chronically and de novo infected cells
Source: J Biol Chem. 2023 Jul 15;299(8):105054. doi: 10.1016/j.jbc.2023.105054 (PMC10432985; doi:10.1016/j.jbc.2023.105054)
Supplement: Supporting Figures S1–S8 [file mmc1.docx]

**Supporting Information**

**Different tau fibril types reduce prion level in chronically and *de novo* infected cells**

Luigi CELAURO^1^, Anna BURATO^1^, Marco ZATTONI^1§^, Elena DE CECCO^1,#^, Marco FANTUZ^2,3^, Federico Angelo CAZZANIGA^4^, Edoardo BISTAFFA^4^, Fabio MODA^4^, Giuseppe LEGNAME^1,*^

^1^Scuola Internazionale Superiore di Studi Avanzati (SISSA), Laboratory of Prion Biology, Department of Neuroscience, Trieste, 34136, Italy

^2^Fondazione per la Ricerca Biomedica Avanzata VIMM, Via Orus 2, 35129 Padova, Italy ^3^Università degli Studi di Padova, Dipartimento di Biologia, Viale G. Colombo 3, 35131, Padova, Italy

^4^Fondazione IRCCS Istituto Neurologico Carlo Besta, Unit of Neurology 5 and Neuropathology, Milan, 20133, Italy

§ current affiliation: Institute of Molecular Regenerative Medicine, Paracelsus Medical University, Salzburg, 5020, Austria

# current affiliation: Institute of Neuropathology, University of Zurich, Zurich, Switzerland

*Corresponding author. Email legname@sissa.it (Prof. Giuseppe Legname)

**This file includes:**

Figures S1-S8

**Supplementary Figure S1**

**
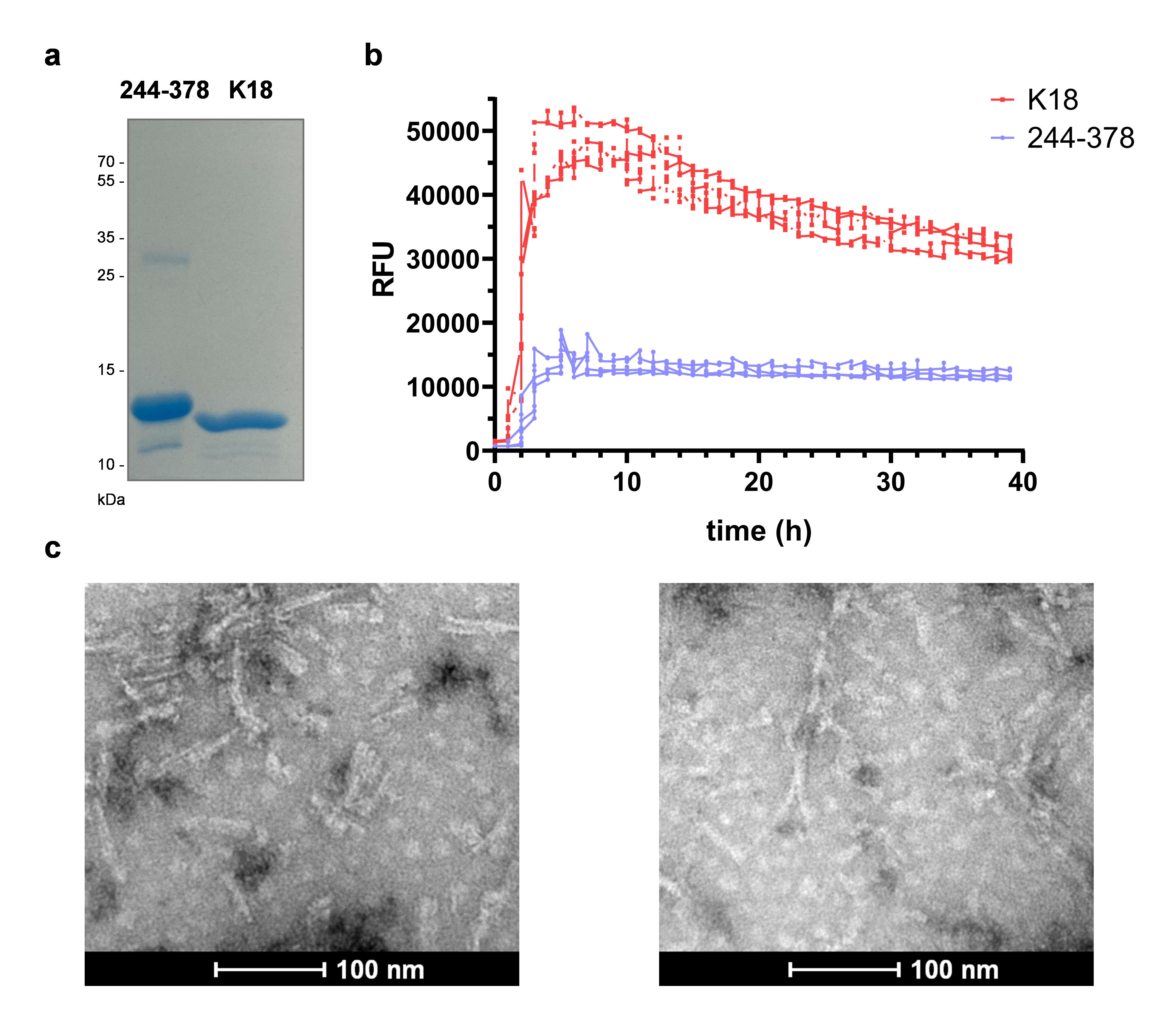
**

**Figure S1. Fibrillization of tau K18 and tau 244-378.**

(a) SDS-PAGE of purified tau 244-378 (~14,5 kDa) and tau K18 (~13,8 kDa). (b) tau K18 and 244-378 *in vitro* fibrillization. Both tau constructs showed a similar aggregation kinetic, with a short lag phase of almost two hours, followed by an exponential phase and a plateau, which was higher in fluorescence values for tau K18. RFU, relative fluorescence unit. (c) TEM images of sonicated K18 (left) and 244-378 (right) tau fibrils.

**Supplementary Figure S2**

**
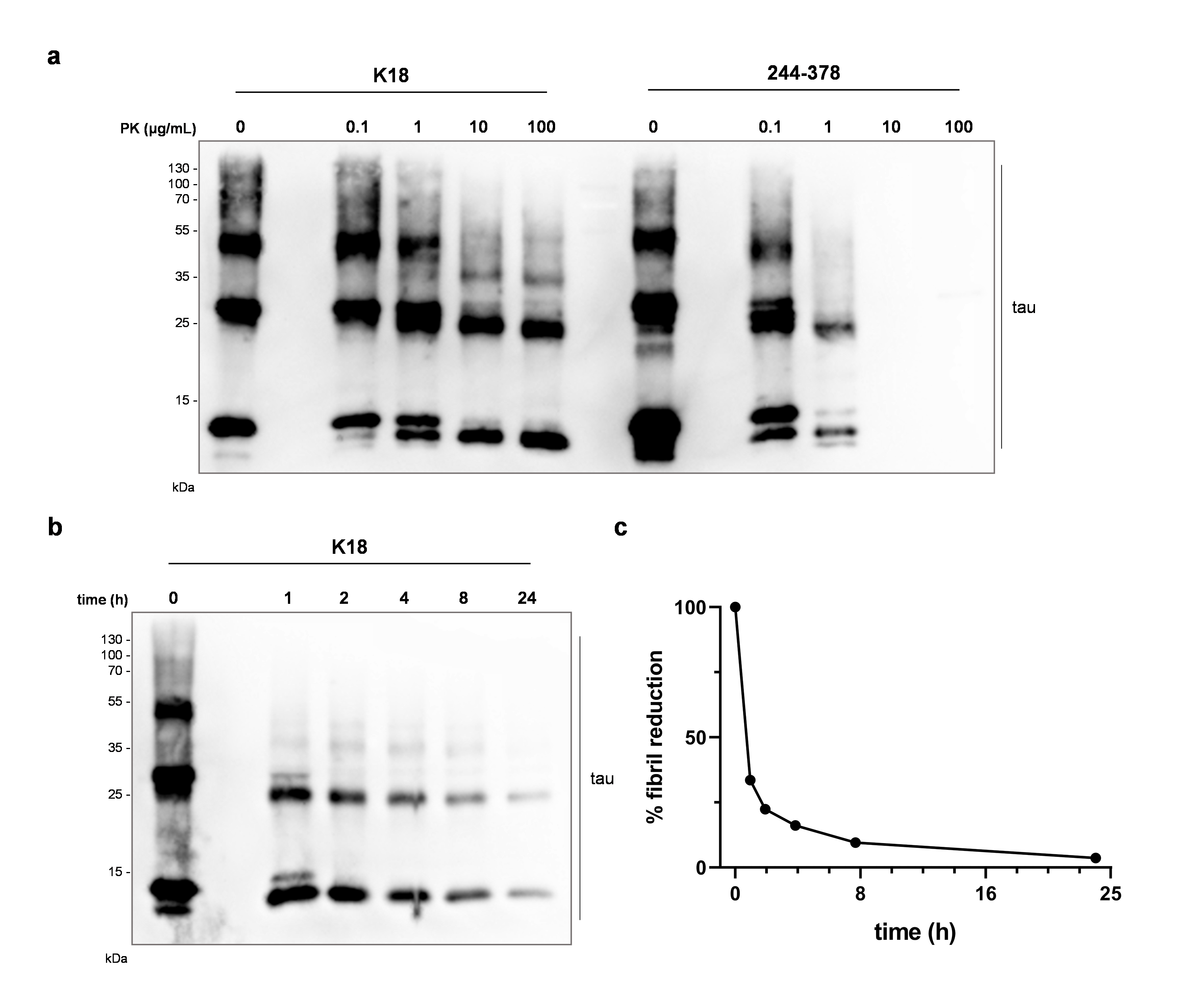
**

**Figure S2. Biochemical characterization of tau K18 and tau 244-378 fibrils.**

(a) WB of 2 μg of tau K18 (left) and 244-378 (right) fibrils after the digestion with 0, 0.1, 1, 10 and 100 μg/mL of PK. (b) WB and (c) graph of 2 μg of tau K18 fibrils digested with 100 μg/mL of PK for 0, 1, 2, 4, 8 and 24h. T_1/2_ 23.46 min.

**Supplementary Figure S3**

**
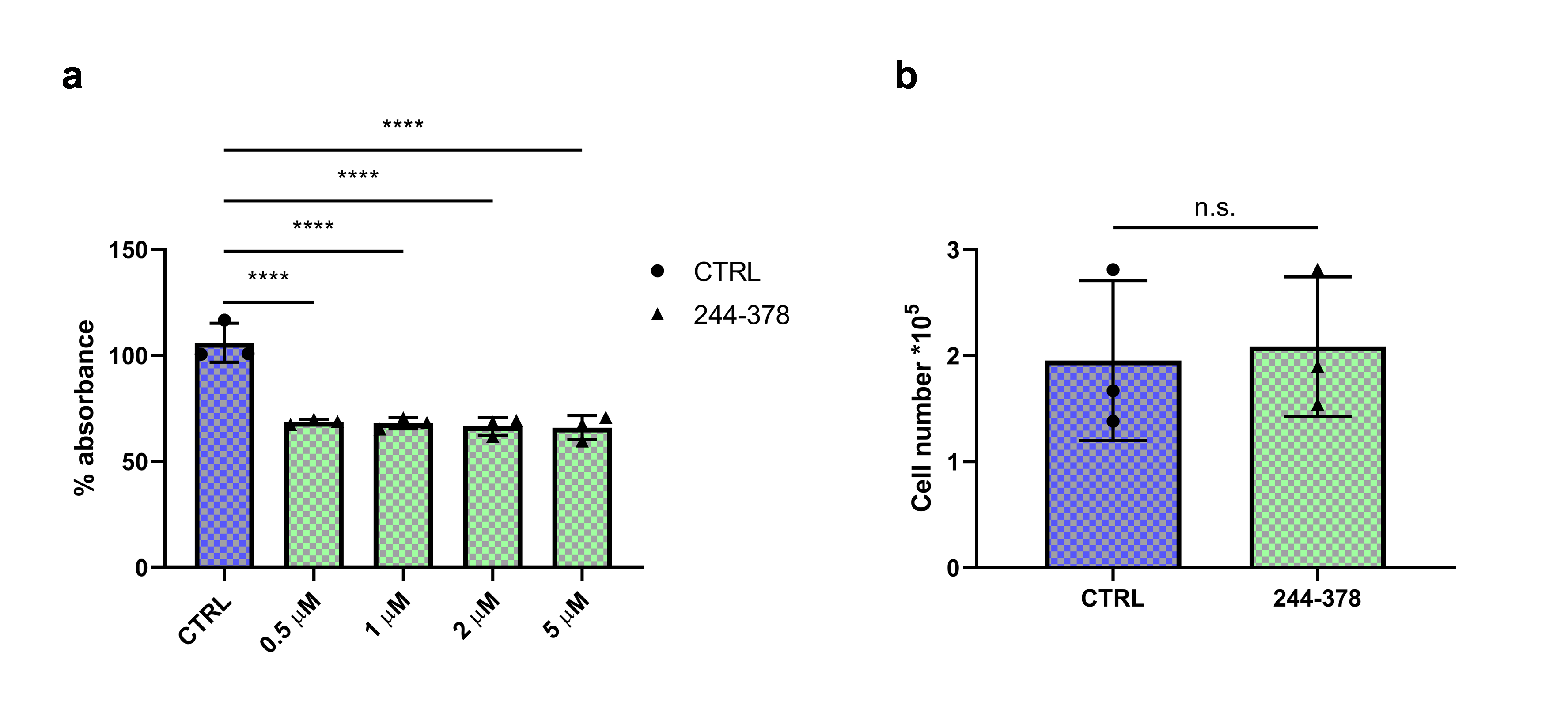
**

**Figure S3. Effects of tau 244-378 fibrils on metabolic activity and viability in ScN2a RML cell line.**

(a) MTT assay of ScN2a RML cell line treated with different concentrations (0.5 μM, 1 μM, 2 μM, 5 μM) of tau 244-378 fibrils for 72h. Data in the graphs are reported as mean ± SD, and each dot represents the mean of six technical replicates with each condition tested in three independent experiments. Data were analyzed with one-way ANOVA with Dunnett’s multiple comparison test: **** p≤0.0001. (b) Cell count analysis of ScN2a RML treated with 2 μM of tau 244-378 for 72h. Data in the graph are reported as mean ± SD. Three independent experiments were performed, and each dot represents the mean of three technical replicates. Data were analyzed with Wilcoxon matched-pairs signed rank test: n.s. not significant.

**Supplementary Figure S4**

**
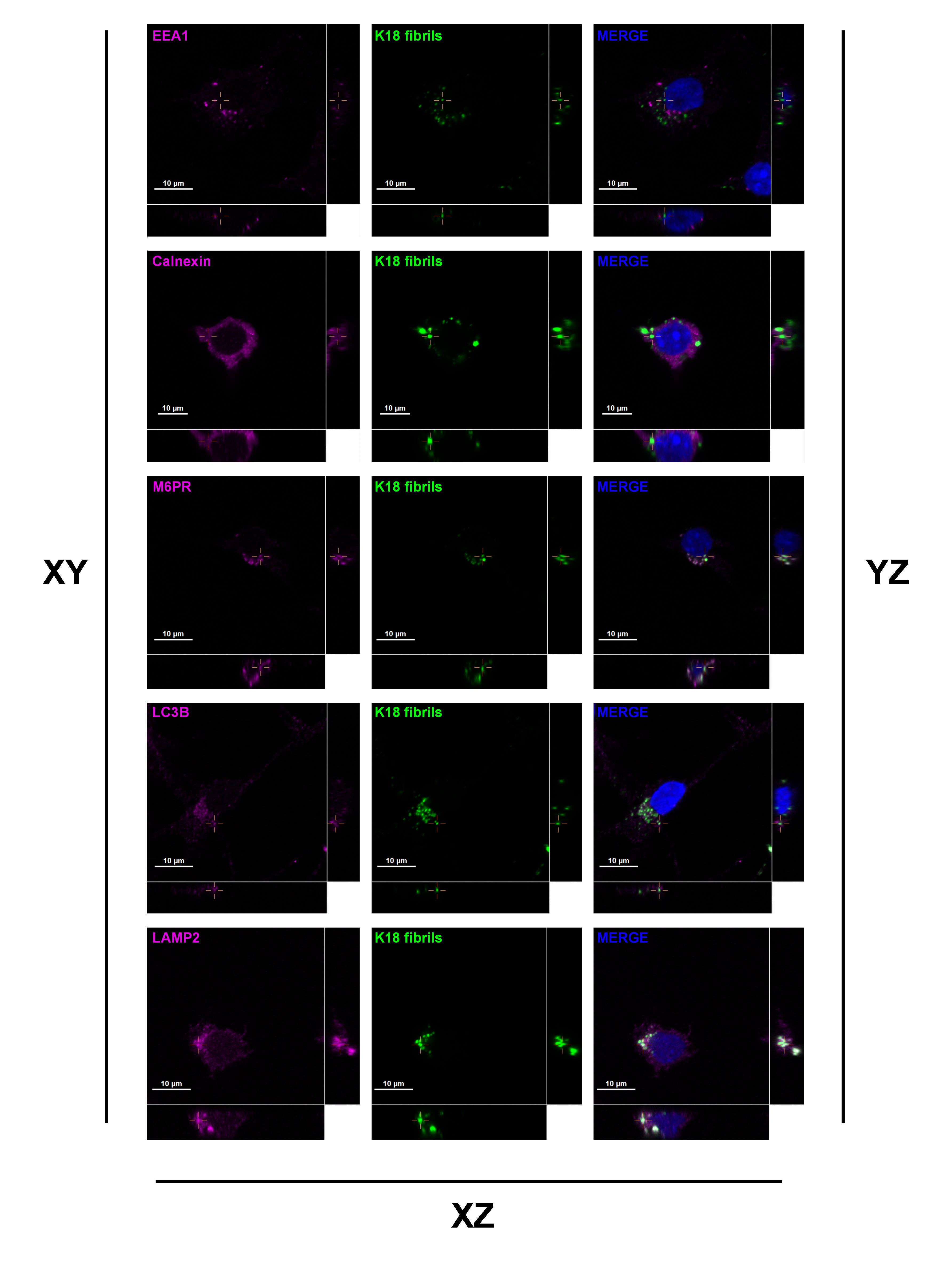
**

**Figure S4. Tau K18 fibril cellular localization.**

ScN2a RML treated for 24h with tau K18-Alexa488 fibrils (green) and stained in magenta for EEA1 (early endosomes), Calnexin (endoplasmic reticulum), M6PR (Golgi apparatus), LC3B (autophagosomes) and LAMP2 (lysosomes). DAPI was used to stain nuclei. Images show one of the central sections of the entire z-stack. All the three orthogonal planes (XY, XZ and YZ) are represented.

**Supplementary Figure S5**

**
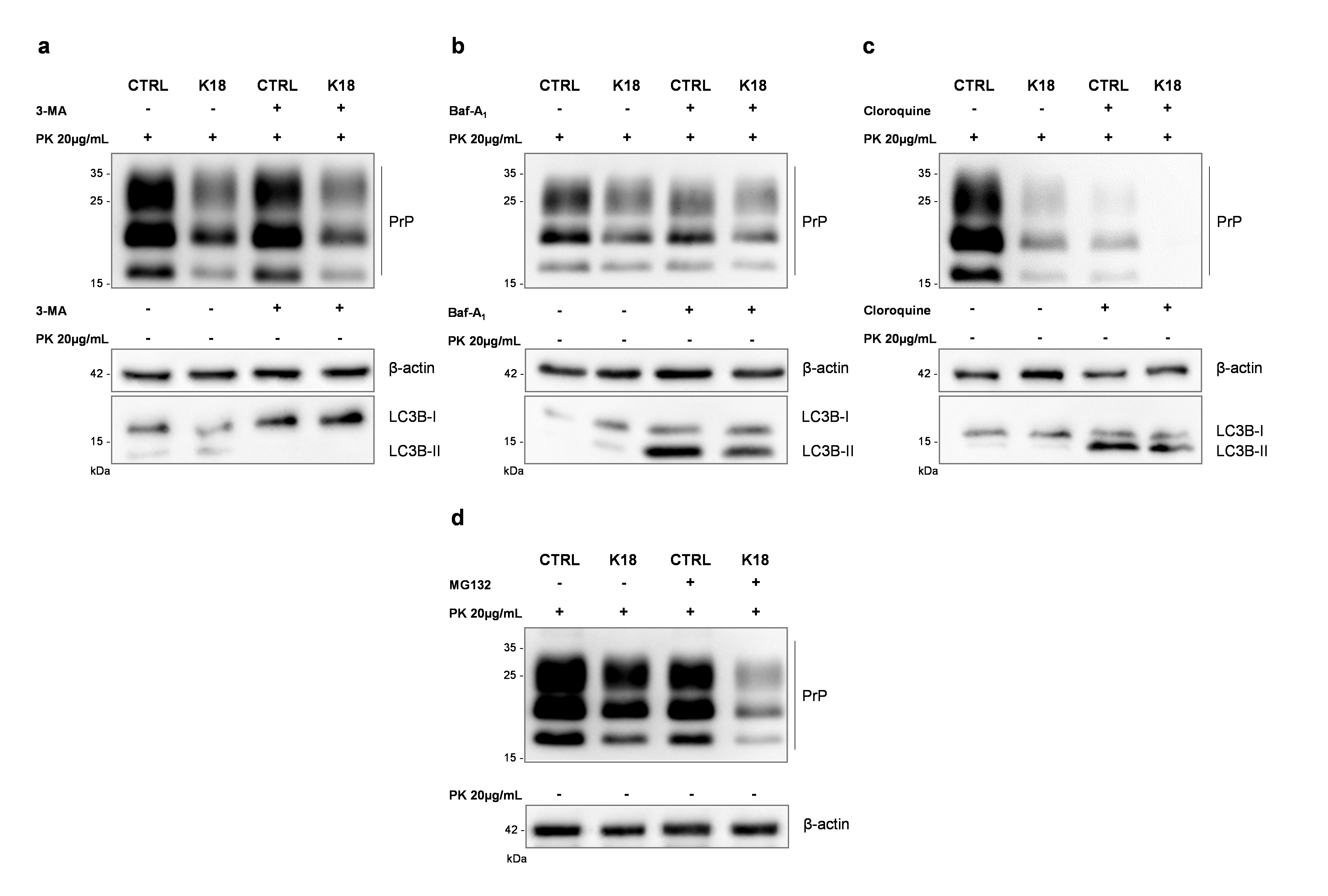
**

**Figure S5. Role of degradation pathways in tau fibrils-mediated PrP^Sc^ clearance.**

Representative WB of ScN2a RML cells incubated for 72h with tau K18 fibrils and treated with (a) 3-methyladenine (3-MA), (b) bafilomycin A_1_ (Baf A_1_), (c) cloroquine and (d) MG132. PK digestion was performed to evaluate PrP^Sc^ levels. β-actin was used as loading control and the amount of LC3B-I and LC3B-II evaluated to confirm the efficacy of the inhibitors.

**Supplementary Figure S6**

**
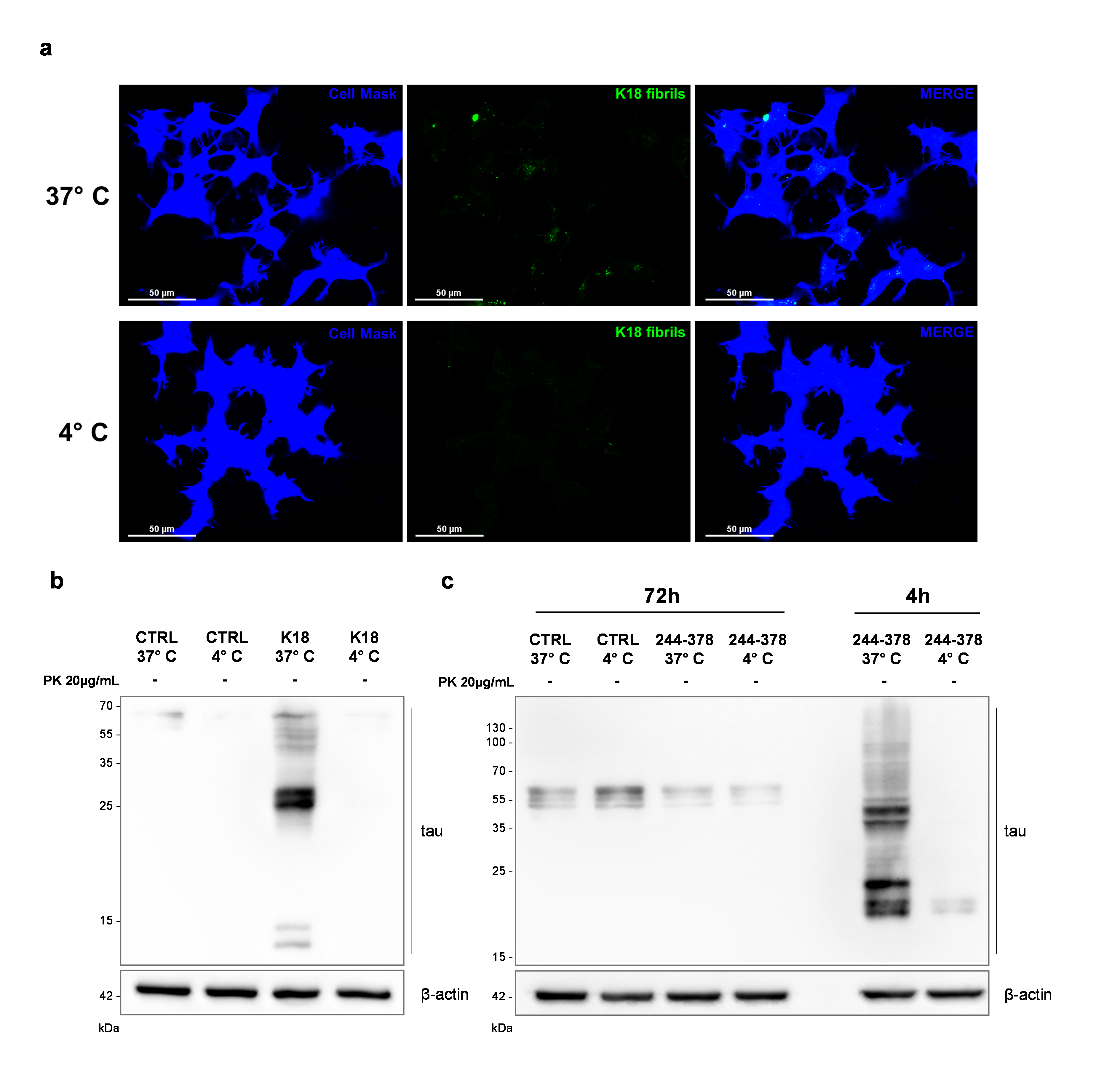
**

**Figure S6. Tau fibril internalization is inhibited at 4° C.**

(a) ScN2a RML cells incubated for 4h with tau K18-Alexa488 fibrils at 37° C (upper panels) and 4° C (bottom panels). Trypan blue was used to quench the fluorescence deriving from extracellular fibrils. Blue CellMask^TM^ was used to stain the entire cell. (b) Representative WB of internalized tau K18 fibrils in ScN2a RML cells at 37° C and 4° C after 4h of incubation with fibrils. Cells were washed with PBS and then left in culture up to 72h without the treatment. Trypsin was used to remove extracellular fibrils before lysis. β-actin was used as loading control (c) Representative WB of internalized tau 244-378 fibrils in ScN2a RML cells incubated with 2 μM of tau 244-378 fibrils for 4h at 37° C or 4° C and then incubated up to 72h without the treatment (left lanes) or immediately lysed (right lanes). Trypsin was used to remove extracellular fibrils. β-actin was used as loading control.

**Supplementary Figure S7**

**
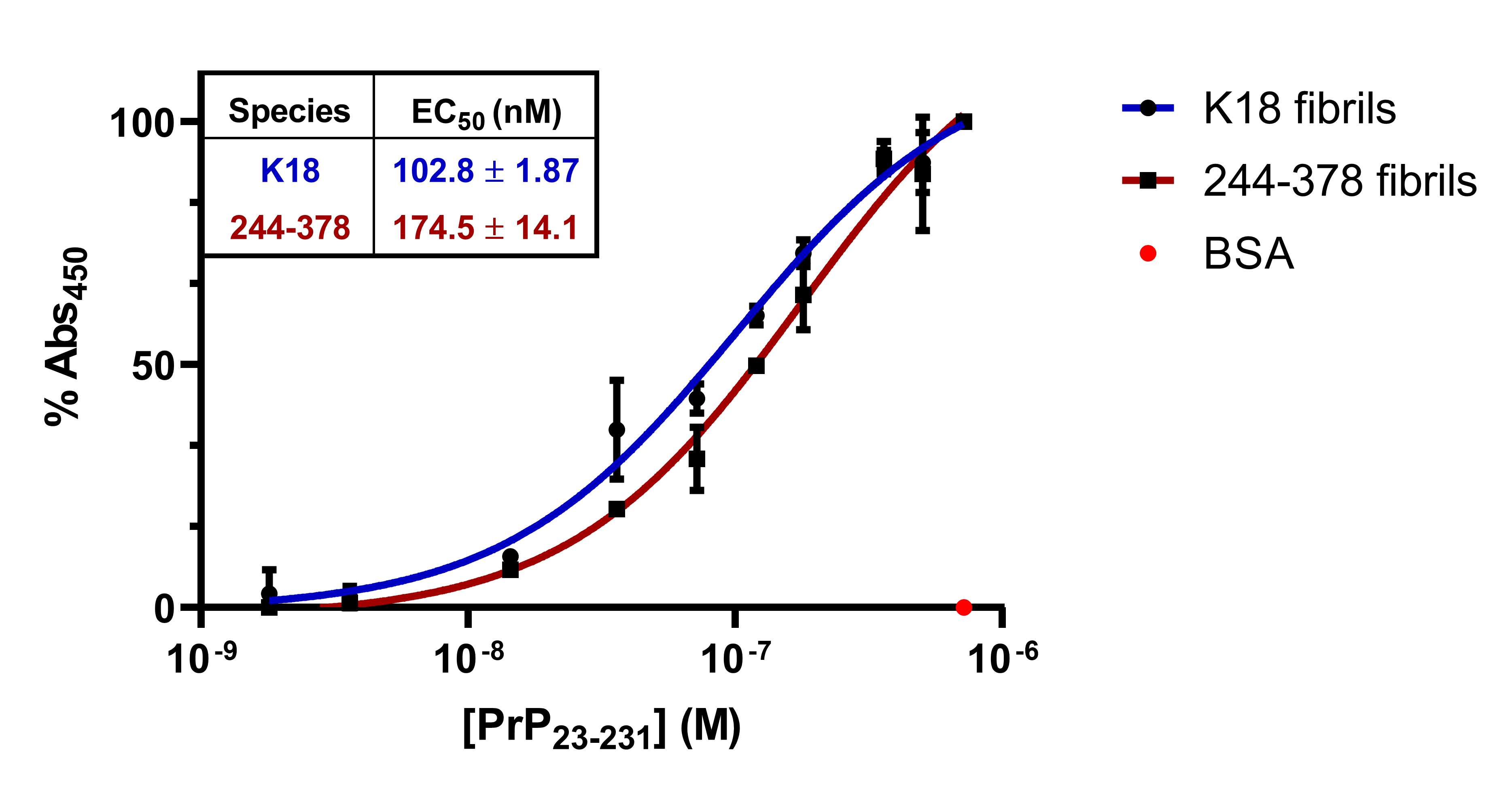
**

**Figure S7. Tau fibrils interact with the recombinant cellular prion protein.**

Binding of recombinant mouse PrP_23-231_ to immobilized tau K18 and 244-378 fibrils was assessed by ELISA assay. Immobilized BSA was used as negative control. Data are shown as mean + SD of a single experiment while EC_50_ values are expressed as the mean ± SD of three independent experiments.

**Supplementary Figure S8**

**
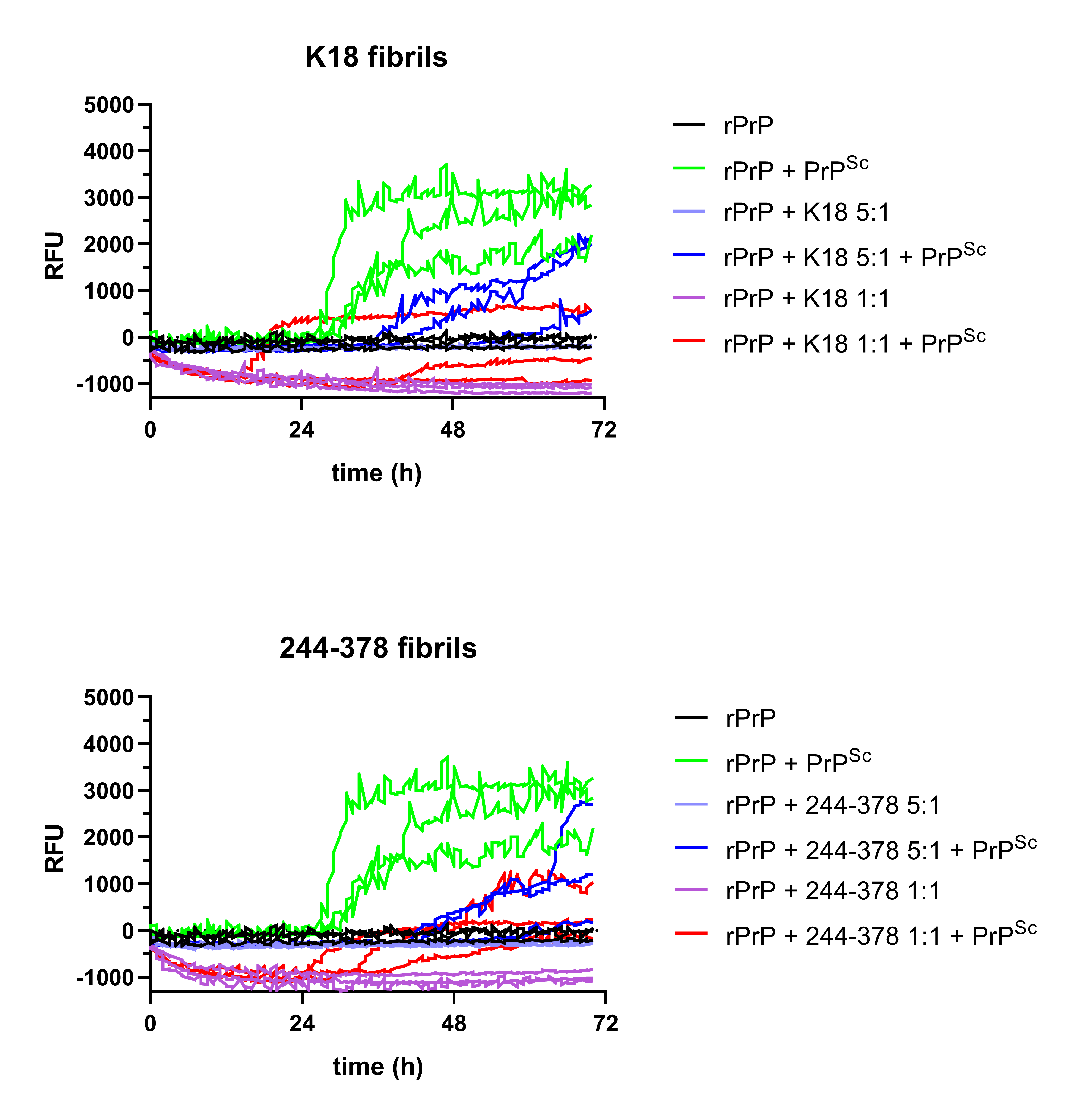
**

**Figure S8. Tau fibril effect on PrP^Sc^-mediated recombinant PrP conversion.**

Effect of K18 (upper panel) and 244-378 (lower panel) tau fibrils on the aggregation of recombinant mouse PrP_23-231_ seeded with 1 ng of PrP^Sc^ through RT-QuIC *in vitro* assay. Recombinant mouse PrP_23-231_ conversion was tested in the presence of 5:1 and 1:1 dilutions of K18 (upper panel) and 244-378 (lower panel) tau fibrils with or without the addition of PrP^Sc^. Each baseline-corrected curve represents the signal of a single well with each condition tested in three technical replicates expressed with the same color. The same trend was confirmed in at least three independent experiments. RFU, relative fluorescence unit.
